# Combining Correlative Cryogenic Fluorescence and Electron Microscopy and Correlative Cryogenic Super‐Resolution Fluorescence and X‐Ray Tomography—Novel Complementary 3D Cryo‐Microscopy Across Scales to Reveal Nanoparticle Internalization Into Cancer Cells

**DOI:** 10.1002/jemt.70071

**Published:** 2025-09-01

**Authors:** Pavitra Sokke Rudraiah, Louisa Herbsleb, Michaela Salakova, Henriette Gröger, Anna Maria Steyer, Frauke Alves, Claus Feldmann, Andreas Walter

**Affiliations:** ^1^ Center for Optical Technologies Aalen University Aalen Germany; ^2^ Institute of Inorganic Chemistry, Karlsruhe Institute of Technology (KIT) Karlsruhe Germany; ^3^ EMBL Imaging Centre European Molecular Biology Laboratory Heidelberg Germany; ^4^ Translational Molecular Imaging Max‐Planck‐Institute for Multidisciplinary Sciences Göttingen Germany; ^5^ Department of Haematology and Medical Oncology, Department of Clinical and Interventional Radiology University Medical Center Göttingen Göttingen Germany

**Keywords:** cryo‐FIBSEM, cryo‐FM, cryogenic correlative light and electron microscopy (cryo‐CLEM), cryogenic correlative light and X‐ray tomography (cryo‐CLXT), cryogenic soft X‐ray tomography (cryo‐SXT), nanoparticle intracellular uptake and trafficking

## Abstract

Understanding the intracellular fate of nanoparticles (NPs) is essential for advancing nanomedicine, particularly in targeted drug delivery for cancer therapy. Here, we present a complementary cryogenic microscopy workflow across scales to investigate the uptake and subcellular localization of zirconyl‐containing inorganic–organic hybrid nanoparticles (IOH‐NPs) in murine breast cancer cells. Our approach integrates cryogenic fluorescence microscopy (cryo‐FM), cryo‐focused ion beam scanning electron microscopy (cryo‐FIBSEM), and cryo‐soft X‐ray tomography (cryo‐SXT), enabling molecular specificity, high‐resolution imaging, and volumetric ultrastructural analysis in near‐native cellular states. We demonstrate that the cryogenic workflow provides enough contrast and resolution across all modalities for quantifying the IOH‐NP uptake: NPs are internalized within 2 h of incubation and progressively accumulate in endolysosomes over time, as confirmed by fluorescence labeling and SXT. Quantitative analysis reveals a marked increase in endolysosomal accumulation of IOH‐NPs from 2 to 24 h. Our findings help to establish multimodal cryogenic microscopy as a powerful tool for nanoscale imaging and quantitative analysis of NP uptake within close‐to‐native cells, offering new insights into NP trafficking and cellular responses relevant to nanomedicine development.

## Graphical abstract

A newly established 3D correlative cryogenic fluorescence microscopy, focused ion beam scanning electron microscopy, and soft x‐ray tomography workflow allowed for enough contrast and resolution to quantify the intracellular uptake of zirconyl‐containing inorganic–organic hybrid nanoparticles as a novel chemotherapeutic drug delivery system into endolysosomal organelles of murine cancer cells.

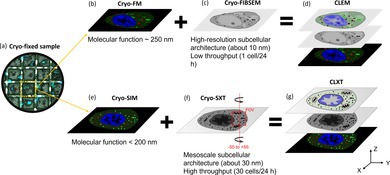

**Summary**

- Multimodal cryogenic microscopy across scales reveals time‐dependent nanoparticle uptake in cancer cells.
- Correlating cryo‐FM with cryo‐FIBSEM and Super‐Resolution cryo‐FM with cryo‐SXT allows for enough contrast and sufficient resolution to study the intracellular distribution of nanoparticles of 60 nm in diameter in 3D.
- Nanoparticles accumulate in endolysosomal compartments over time and enter murine breast cancer cells within 2 h.
- Quantitative cryo‐SXT confirms increasing endolysosomal localization of nanoparticles from 2 to 24 h.
- The established workflow offers near‐native imaging of nanoparticle–organelle interactions with higher throughput compared to room temperature.

## 1 Introduction

### 1.1 Nanoparticles (NPs)as a Drug Delivery System for Chemotherapeutics

NPs have emerged as promising tools in biomedical research, particularly in drug delivery, imaging, and cancer theranostics. Their ability to be taken up by cells and interact with intracellular organelles is crucial in determining their therapeutic efficacy and potential cytotoxic effects. Understanding the internalization pathways, intracellular trafficking, and fate of NPs within cells is essential for optimizing their biomedical applications (Duncan and Richardson 2012; Zhang et al. 2008). Endocytosis is a primary mechanism through which cells take up NPs. This process encompasses various pathways, including clathrin‐mediated, caveolae‐mediated, and macropinocytosis, each dictating different intracellular fates for the NPs (de Almeida et al. 2021; Wang and Wang 2023). Lysosomal involvement has gained significant attention among these pathways due to its role in NP degradation and cellular responses. The lysosome, a membrane‐bound organelle rich in hydrolytic enzymes, serves as a key compartment for the processing and recycling of internalized materials (Tian et al. 2022). The interaction of NPs with lysosomes can influence cellular homeostasis, triggering changes in lysosomal size, activity, and overall function, potentially leading to cellular stress and altered metabolic pathways (Wang et al. 2019).

Here, we investigate the cellular uptake of zirconyl‐containing inorganic–organic hybrid nanoparticles (IOH‐NPs) as a novel chemotherapeutic drug delivery system developed and described elsewhere (Heck et al. 2015). These IOH‐NPs are characterized by an organic drug or dye anion and an inorganic cation to obtain a saline composition that is insoluble in water (Heck et al. 2015). Typical examples are [ZrO]^2+^[GMP]^2−^ or [ZrO]^2+^[FMN]^2−^ IOH‐NPs that consist of chemotherapeutic gemcitabine monophosphate anions ([GMP]^2−^) or luminescent flavinmononucleotide ([FMN]^2−^) anions, respectively, and zirconyl ([ZrO]^2+^) cations (Heck et al. 2015). Specific features and advantages of the IOH‐NPs, initially developed at the Karlsruhe Institute of Technology (Roming et al. 2008), are: (i) synthesis in water, (ii) high load of drug or fluorescent dye (up to 85 wt‐% of total NP weight), (iii) high photostability, (iv) intense emission, (v) flexible NP composition, and (vi) platform concepts to be used for many drugs and applications. Specifically, the therapeutic benefits of the high drug load in minimizing undesired side effects and overcoming mechanisms of chemoresistance have been demonstrated in a pancreatic cancer mouse model (Ischyropoulou et al. 2023). Due to the presence of [ZrO]^2+^ (and hence the higher absorption of X‐rays) and the here applied [ZrO]^2+^[(CMP)_0.99_(DUT647)_0.01_]^2−^ IOH‐NPs (CMP: cytidine monophosphate), also containing the red‐emitting fluorophore Dyomics‐647‐uridine triphosphate DUT647, the IOH‐NPs (with a diameter of about 60 nm) are visible and detectable using cryogenic fluorescence microscopy (cryo‐FM), electron microscopy (EM), and soft X‐ray microscopy (SXT). Due to their fluorescence labeling, IOH‐NPs can also be optically monitored in vivo and ex vivo over time, confirming their accumulation at inflammatory sites in the lungs (Napp et al. 2018) and within tumor tissue (Ischyropoulou et al. 2023) in previous studies. To understand the fundamental concepts of how these IOH‐NPs interact with the biological systems at cellular and subcellular levels, how physicochemical properties influence intracellular trafficking, and how this might affect nanomedicine efficacy, especially in tumor cells, there is a high need to visualize intracellular internalization and trafficking at a single‐cell level. We propose and highlight correlative cryogenic microscopy across scales and modalities as a powerful and novel tool for these investigations.

### 1.2 Correlative Cryogenic Microscopy

An important goal in cell biology and biomedical diagnostics is to image specific molecules directly within their native cellular context. This requires sample preparation and microscopy techniques that allow both the visualization of the subcellular architecture of a cell and, at the same time, the unambiguous localization of macromolecular complexes within this cell. To gain such insights, imaging technologies need to be combined and correlated. For the best functional and structural characterization of macromolecules, the ideal imaging setup provides protein‐specific information without interfering with the native structure of the cell and covers the relevant resolution range from micrometers to nanometers. An appropriate tool to approach this goal is correlative cryogenic microscopy. While fluorescence microscopy (FM) provides high molecular specificity, it lacks information about surrounding structures. This ultrastructural context can be provided by EM at nanometer resolution or, with higher throughput at the expense of resolution (up to 30 nm), using SXT.

To correlate data from FM and EM or SXT, the subcellular structure of the specimen imaged using both modalities must be identical. This requires the specimen to be preserved and the molecules and subcellular structures to be locked in position. Since cells are plunge‐frozen and vitrified for cryo‐SXT and cryo‐EM in a close‐to‐native state, it is evident that the specimen can also be cryopreserved for FM and then imaged sequentially. In addition to compatibility with SXT and EM, there are several reasons why it is beneficial to image fluorescence signals at cryogenic temperatures: Spectral properties are improved as the emission spectra of many fluorophores are narrower at cryogenic temperatures compared to room temperature (Kaufmann et al. 2014; Moerner and Orrit 1999). The working lifetime of fluorophores is increased by a factor of 30 since photobleaching is significantly reduced at cryogenic temperatures (Schwartz et al. 2007). This simplifies the quantification of the fluorescent signal over time, dramatically improves the signal‐to‐noise ratio (SNR), and allows for prolonged and repeated exposure.

Compared to cryogenic conditions, EM and X‐ray imaging at room temperature often require extensive sample preparation steps, including chemical fixation, dehydration, and staining. These processes can introduce structural artifacts and compromise the integrity of biological samples. Cryogenic microscopy techniques offer a significant advantage by preserving biological specimens in a vitrified, near‐native state, thereby providing high‐resolution insights into the cellular ultrastructure and molecular organization at nanometer resolution (Mahamid et al. 2015; Villa et al. 2013).

**FIGURE 1:**
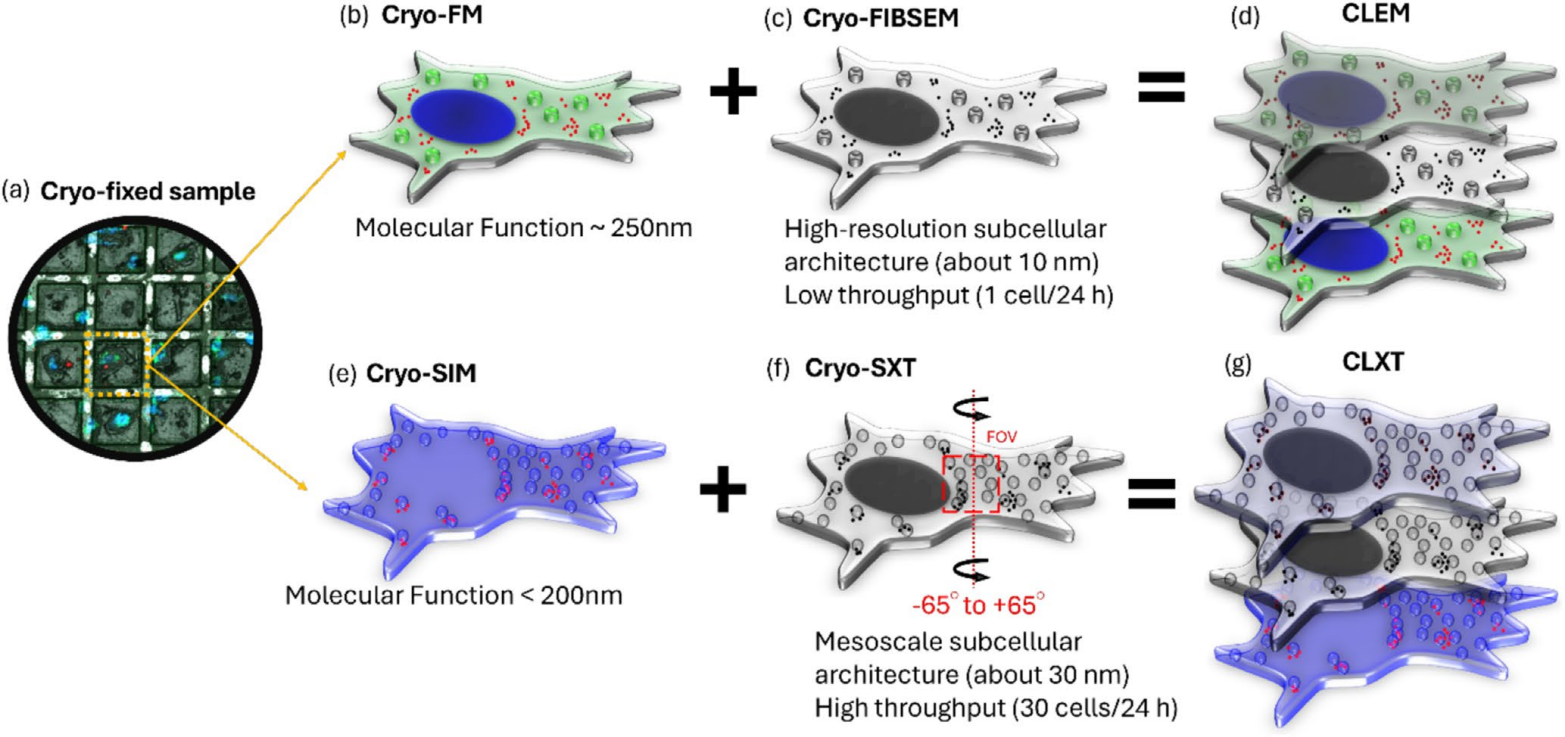
Schematic of cryogenic 3D CLEM (a–d) and CLXT (a–g) workflows combining FM with nanoscale FIBSEM or with SXT. (a) Cryo‐fixed cell monolayer on an EM finder grid. (b) FM image of the cell staining highlighting the nucleus (blue), lipid droplets (green), and IOH‐NPs (red). (c) The same cell is imaged using nanoscale FIBSEM. (d) Both cryo‐FM and cryo‐FIBSEM provide 3D information on entire single cells. Here we show a 3D projection of both modalities as an overlay of FM and FIBSEM images for correlation. (e) SIM image of the cell staining highlighting lysosomes (blue) and IOH‐NPs (red). (f) The same cell with an indicated field of view (FOV) is imaged using soft X‐ray tomography at angles from −65° to +65° (or less). (g) Both cryo‐SIM and cryo‐SXT provide 3D information of entire single cells. Here we show a 3D projection of both modalities as an overlay of SIM and SXT images for correlation.

In this study, we employ a multimodal cryogenic microscopy approach across scales that integrates cryogenic FIBSEM, cryogenic SXT, and cryogenic FM, including structured illumination microscopy (SIM) and Airyscan microscopy, to investigate the internalization and intracellular fate of IOH‐NPs in mouse breast cancer cells. As shown in Figure 1, we combine two separate cryogenic workflows in three dimensions (3D): (1) Correlative light and electron microscopy (cryo‐CLEM) by combining FM and FIBSEM, and (2) correlative light and X‐ray tomography (cryo‐CLXT) by combining SIM and SXT. Here, cryo‐FIBSEM enables the 3D imaging of the cellular architecture by precisely milling vitrified samples and capturing high‐resolution cross‐sections, preserving the organelle morphology and NP distribution with minimal preparation artifacts (Sartori et al. 2007; Schaffer et al. 2015). Cryo‐SXT, a complementary label‐free imaging technique, provides whole‐cell imaging in 3D at a resolution of about 30 nm, allowing for the visualization of NP uptake and trafficking across different cellular compartments without the need for staining or sectioning (Chiappi et al. 2016). Meanwhile, cryogenic super‐resolution FM, specifically cryo‐SIM and cryo‐Airyscan, enhances optical resolution beyond the diffraction limit, enabling the precise localization of fluorescently labeled NP and their interactions with subcellular structures (Marshall et al. 2024; Wu et al. 2020).

By integrating these cryogenic microscopy techniques, we visualized the subcellular distribution of IOH‐NPs in murine breast cancer cells over time. Our findings reveal that IOH‐NPs enter cells within 2 h of exposure, followed by progressive lysosomal internalization, leading to an increase in lysosomal size over time. This study highlights cryogenic microscopy's transformative potential in biomedical research and advances our understanding of NP‐cell interactions. The ability to visualize nanoscale structures in their close‐to‐native state and their correlation with molecular markers and fluorescently labeled IOH‐NPs localized by high‐resolution FM represents a significant step forward in both nanomedicine and structural cell biology.

Figure 1 illustrates the bi‐headed cryo‐CLEM and cryo‐CLXT workflows, integrating FM with EM or SXT techniques. This approach employs a grid‐based imaging strategy in which breast cancer cells are initially identified using FM ([b] and [e] [cryo‐FM and SIM]). A magnified region highlights a single cell selected for further investigation. The schematic fluorescence image of the cell shows specific markers that were used for the respective workflows, providing molecular and functional insights: the nucleus (blue), IOH‐NPs (red), and lipid droplets (green) for cryo‐CLEM, and IOH‐NPs (red) and lysosomes (blue) for cryo‐CLXT. The same cell is imaged either using FIBSEM for high‐resolution ultrastructural context (of about 10 nm) or using SXT to obtain mesoscale structural information of about 30 nm in resolution and, at the same time, high throughput ([c] and [f]). The grayscale images represent the ultrastructural details captured by these techniques. The top‐right sections of Figure 1d,g demonstrates the overlay of different microscopy modalities for multimodal analysis. Finally, 3D tomography and image stacking are performed and integrated with the fluorescence data, enabling a comprehensive visualization and understanding of the cellular architecture in 3D and the localization of the IOH‐NP within these complex intracellular structures.

## 2 Materials and Methods

### 2.1 Sample Preparation for Correlative Cryo‐Microscopy

#### 2.1.1 Cell Culture

Basal‐like breast cancer (BLBC) cells, murine H8N8 cells with tumor stem cell properties, that were developed from bi‐transgenic WAP‐T/WAP‐mutp53 tumors, were maintained in high glucose (4.5 g/L d‐Glucose) Dulbecco's modified Eagle's medium (DMEM) containing 10% fetal calf serum (FCS, all from Thermo Fisher Scientific Corp., Waltham, MA) at 37°C in a humidified atmosphere with 5% CO_2_.

#### 2.1.2 Hydrophilization of EM Grids

Both 200‐mesh gold grids with a SiO_2_ support film (R1.2/20, Quantifoil) and 100 holey carbon film support gold grids (Au G200F1 finder, R2/2) were used for the experiment. The EM grids were dipped in 70% ethanol in a laminar flow hood for initial cleaning. EM grids were then plasma cleaned for 2 min using a 72:25 argon/oxygen mixture and then coated with 50 μL of fibronectin at a concentration of 50 μg/mL for 30 min on a parafilm‐coated coverslip, which promotes proper cell growth on the grids. After coating, the grids were transferred to a culture dish and washed twice with PBS (phosphate‐buffered saline).

#### 2.1.3 Cell Seeding and Incubation

After 24 h of hydrophilization, the EM grids were transferred to 6‐well multidishes with 3–4 grids per well. Approximately 200,000 H8N8 cells were seeded into each well. After 2 h, the medium was changed to remove unattached cells and was incubated for 24 h for proper cell attachment. After an overnight incubation for proper cell attachment, IOH‐NPs were added to the cells. Brightfield microscopy that was performed after the incubation confirmed that the cells were in good condition.

#### 2.1.4 NPs Incubation

Based on previous experimental data, for the CLEM workflow, NP incubation times of 2 and 4 h were chosen. For the CLXT workflow, incubation times of 2, 6, and 24 h were used. Fluorescently labeled, water‐soluble IOH‐NPs with an approximate size of ~60 nm, [ZrO]^2+^[(CMP)_0.99_(DUT647)_0.01_]^2−^, were employed. CMP refers to cytidine monophosphate, and DUT647 is a Dyomics fluorophore excited at 651 nm and emitting at 673 nm. The IOH‐NPs were prepared at a concentration of 10 μg/mL and added at 20 μL per well to the respective wells containing cell‐seeded EM grids for the selected incubation times (for CLEM workflow: 2 and 4 h; CLXT workflow: 2, 6, and 24 h). Each chamber contained four EM grids.

#### 2.1.5 Addition of Markers for Correlation Studies

Thirty minutes before plunge freezing, cells were labeled to enable correlation within the CLEM and CLXT workflow. The following labels were used for each workflow:

1. *CLEM workflow*: Cells were labeled with Hoechst (blue) at a concentration of 5 μM to visualize nuclei and BODIPY (green) at a concentration of 5 μM to track lipid droplets, enabling correlation between cryogenic FM and FIBSEM.
2. *CLXT workflow*: Cells were stained with LysoTracker Blue at a concentration of 200 nM for lysosomal tracking. Additionally, nonfluorescent fiducial gold NPs with a concentration of 2 μL, with a diameter of approximately 250 nm, were added to facilitate precise correlation between cryogenic SIM and SXT datasets.

#### 2.1.6 Plunge Freezing

EM grids with cells incubated with IOH‐NPs, along with their respective control grids for each time point, were plunge‐frozen with blotting times of 4 s for CLEM workflow and 1 s for CLXT workflow using the Leica EM GP2 plunge freezer (Leica, Wetzlar, Germany). In the CLXT workflow, gold fiducial markers were added right before plunge freezing to each grid (Okolo et al. 2022).

### 2.2 Cryogenic CLEM Workflow

#### 2.2.1 Cryo‐LSM 900 With Airyscan 2 Microscopy

Vitrified samples were imaged using the Cryo‐LSM 900 microscope with an Airyscan 2 detector (Zeiss), equipped with a cryo‐stage (Linkam Scientific, Salfords, UK) and objectives of 5×, 10×, and 100× to capture high‐resolution fluorescence data. Transmitted and reflected light images were captured using various fluorescent channels at wavelengths of 405, 488, and 647 nm. A brightfield mosaic scan was initially acquired to identify regions of interest (ROI) and to verify proper sample vitrification and structural integrity, based on the absence of crystalline ice and the presence of smooth vitreous ice features. Fluorescence imaging was performed with multiple channels recorded simultaneously to enhance signal detection. 3D image stacks were collected along the Z‐axis, enabling volumetric reconstruction of cellular structures. Airyscan 2 technology facilitated super‐resolution imaging by improving SNR and spatial resolution. Postacquisition processing, including alignment and noise reduction, was conducted using Zeiss Zen software. Chromatic aberration corrections and data refinement were applied to ensure high‐fidelity reconstruction of the fluorescence signal.

#### 2.2.2 Cryo‐FIBSEM Imaging

At first, the frozen cells were imaged using the LSM900 Airyscan 2 (Zeiss, Oberkochen, Germany). After obtaining the cryo‐FM images, the frozen samples were transferred to the Crossbeam 550 (with Quorum cryo‐stage) via the Zeiss correlative cryo‐CLEM holder. Then, the cells were targeted using Zen Blue Connect (Zeiss, Oberkochen, Germany), which overlaid images from the light and scanning EMs. Before milling, the sample surface was coated with a thin layer of inorganic platinum through sputter coating to minimize charging and a thick layer of organic platinum to reduce ion beam‐induced damage. Data were collected at 2.3 kV and 50 pA, with a pixel size of 10 nm (in both X and Y directions) and a 30 nm distance between individual slices, utilizing the SE2 detector. The images were acquired with a dwell time of 0.2 μs and a line averaging of 59. While iterating between milling and imaging, a focused gallium ion beam (FIB) with a voltage of 30 kV and a current of 300 pA was employed. Either a relatively shallow cutting angle of 10° was chosen to achieve a larger cross‐section through the cell, or an orthogonal cross‐section through the cell. Due to the low SNR ratio and a higher number of artifacts in the cryo‐FIBSEM datasets, postprocessing on the data was carried out. A variational stationary noise removal (Fehrenbach et al. 2012), Gaussian blur, and a local contrast enhancement (CLAHE) were performed.

### 2.3 Cryogenic CLXT Workflow

#### 2.3.1 Cryo‐3D SIM Imaging at Diamond Light Source

Vitrified samples were loaded into a Linkam cryostage (Linkam Scientific, Salfords, UK) coupled to the cryo‐SIM microscope at beamline B24 for SIM data collection (Phillips et al. 2020). A brightfield mosaic scan was initially acquired to identify ROI and to verify proper sample vitrification and structural integrity, based on the absence of crystalline ice and the presence of smooth vitreous ice features. Fluorescently labeled cells were then excited in pairs using two laser sources—405 nm (blue) and 647 nm (far red) – with exposure times of 100 and 50 ms (depending on the staining efficiency) using a 100X 0.9 NA objective lens with a working distance of 2 mm. Fluorescence data were recorded through 452 and 655 nm filters across two detection channels. In cryo‐SIM imaging mode, each Z‐slice was recorded 15 times (five phases for each three angles in the structured illumination pattern) at increments of 125 nm along the Z‐axis. Images were captured at a resolution of 512 × 512 pixels with a pixel size of 125 nm. These images were then reconstructed into 1024 × 1024 pixel image stacks with a voxel size of 62.5 × 62.5 × 125 nm. Both brightfield and structured illumination images were collected as Z stacks to facilitate 3D reconstruction. The raw SIM data were processed using optical transfer function (OTF) files in SoftWoRX 6.5.2. Chromatic aberration between different wavelength channels was corrected, and image alignment was refined using Chromagnon software.

#### 2.3.2 Cryo‐SXT Imaging and Reconstruction at Diamond Light Source

Following cryo‐SIM imaging, the vitrified samples were transferred to a transmission X‐ray microscope (TXM; Carl Zeiss Microscopy Inc.) at beamline B24 under high vacuum conditions (10^−6^ Torr) (Kounatidis et al. 2020). The grids were first examined using a low‐resolution visible‐light microscope to verify alignment and locate the region of interest identified in cryo‐SIM imaging. A 2D X‐ray mosaic of each region of interest was then acquired to refine the selection of specific 10 × 10 μm areas for high‐resolution 3D imaging. Then the 3D cryo SXT data were collected as a tilt series spanning −65° to +65° in 0.2° increments, with exposure times ranging from 0.5 to 1 s per step. The automatic tomographic reconstruction was done using computational methods such as the simultaneous iterative reconstruction technique (SIRT), weighted back projection (WBP), or patch tracking via Batchruntomo within the IMOD framework (Harkiolaki et al. 2022; Mastronarde and Held 2017). Manual alignment and reconstruction were conducted using IMOD if automatic reconstruction was insufficient.

### 2.4 Correlation and Image Analysis

**FIGURE 2:**
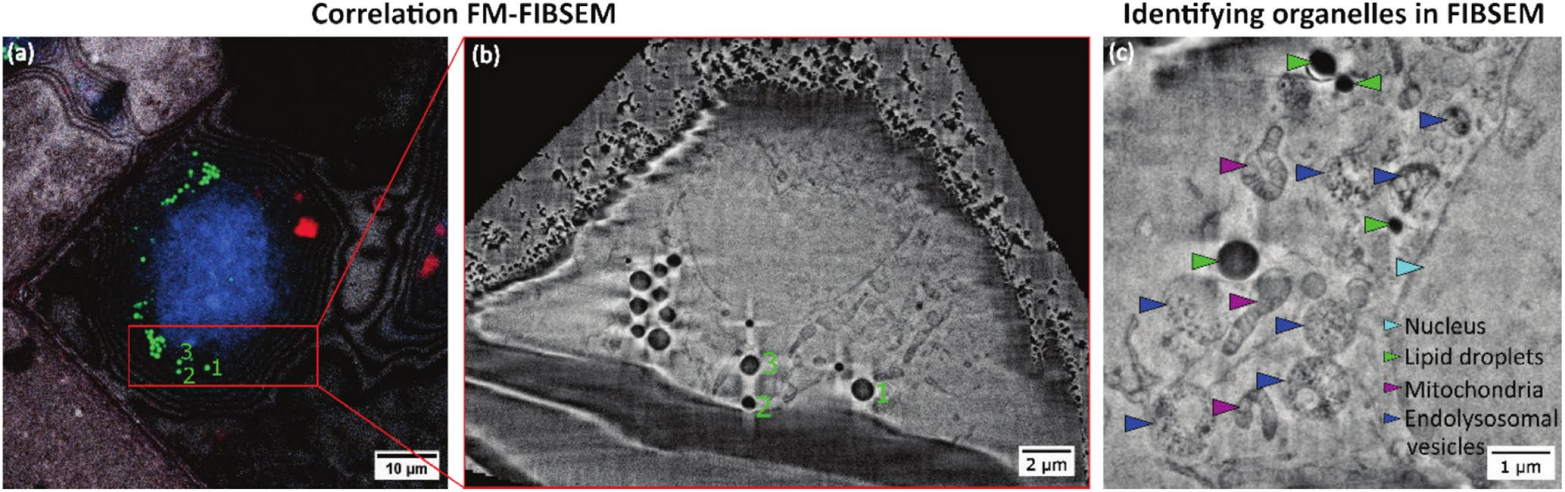
Correlative Cryogenic FIBSEM can be aligned based on lipid droplets and provides enough contrast to visualize the ultrastructural context. (a) and (b) highlight the correlation between FM and FIBSEM based on lipid droplets. Individual lipid droplets (green in FM) can be relocated in FIBSEM datasets due to their electron‐dense, round shape. Lipids that belong together are marked with numbers from 1 to 3 and enable a prealignment. (c) shows that various organelles can be identified in cryo‐FIBSEM datasets, including mitochondria (magenta arrowheads), endolysosomal vesicles (blue), lipid droplets (green), and the nucleus (cyan).

1. *CLEM workflow*: For correlation between Cryo‐FM and FIBSEM, lipid droplets are used, since they can be identified within both modalities, as shown in Figure 2a,b. By analyzing both images side by side, single lipid droplets can be allocated across modalities, and NPs can be identified. The protocol is described elsewhere (O'Connor et al. 2024). A total of three cells were visually inspected.
2. *CLXT workflow*: Image registration and correlation between cryo‐SIM and cryo‐SXT datasets are achieved using nonfluorescent fiducial markers (e.g., ~250 nm gold nanospheres), fluorescently labeled IOH‐NPs, grid coordinates, and intrinsic cellular features such as nuclei and organelle boundaries to ensure precise spatial alignment. This process is facilitated by software tools including eC‐CLEM, Amira, IMOD, ChimeraX, and custom‐developed pipelines at B24, as detailed elsewhere (Kounatidis et al. 2020). Following this multimodal correlation, IOH‐NPs detection was carried out using the Difference of Gaussian (DoG) algorithm (blob_dog function) from the *scikit‐image* library (Van der Walt et al. 2014). This method identifies bright, circular blobs within a specified size range (min_sigma = 5, max_sigma = 6) in volumetric images with a dark background, using an intensity threshold of 0.05. Since the input images featured a white background and dark IOH‐NPs, we first enhanced the contrast by applying contrast‐limited adaptive histogram equalization (CLAHE) on the grayscale images. The contrast was then inverted using a Bitwise NOT operation. These preprocessing steps enabled robust detection of local intensity maxima across multiple spatial scales, facilitating automated identification of IOH‐NP‐like bright spots within the complex cellular ultrastructure. A total of 17 cells were analyzed across the time points: five cells at 2 h, five cells at 6 h, and seven cells at 24 h.

## 3 Results

### 3.1 Cryogenic Microscopy Across All Modalities Provides Enough Contrast and Resolution to Study NP Uptake Into Close‐To‐Native Cancer Cells

Here, we present the combination of cryo‐CLEM and cryo‐CLXT as a higher‐throughput alternative to 3D CLEM at room temperature. By plunge freezing the cancer cells at different time points after IOH‐NP uptake, we were not limited by the prolonged fixation and staining procedures for room‐temperature EM of up to 1 week, but we were able to image cancer cells directly after the plunge freezing, which took less than 2 h. Here, we show that lipid droplets can be used to align the images between FM and FIBSEM (Figure 2). We have previously demonstrated that this is also a valid strategy for the correlation between FM and SXT datasets, and allows for the automation of the process (O'Connor et al. 2024). For the particular CLXT dataset shown below, fiducial markers and IOH‐NPs were used for the correlation of the two modalities. Figure 2 highlights this correlation approach between FM and FIBSEM. The distribution of lipid droplets in FM (green) can be relocated in FIBSEM. As highlighted in Figure 2c, cryogenic FIBSEM can be used as a 3D‐EM stand‐alone technique (in combination with FM) to study NP uptake since it provides enough contrast to identify different organelles within the cell, including endolysosomal vesicles, mitochondria, the nucleus, and lipid droplets.

The imaging throughput was additionally increased by integrating cryo‐SXT into the workflow. Single‐cell soft X‐ray tomograms typically require acquisition times between 30 min and 1 h, depending on the sample thickness. Compared to other volume imaging techniques used to analyze cellular ultrastructure, such as FIBSEM, this approach enables the generation of high‐throughput data suitable for quantitative statistical analysis. While FIBSEM can achieve a resolution of up to ~10 nm, acquiring a full single‐cell dataset at this level takes up to 48 h, during which the sample is irreversibly destroyed. In contrast, SXT allows for subsequent targeted high‐resolution imaging using techniques such as transmission electron microscopy (TEM) or FIBSEM at both cryogenic and room temperatures. In addition to the higher throughput, cryogenic microscopy, in contrast to microscopy based on chemical fixation, also allows for preserving the native structure of single cells.

**FIGURE 3:**
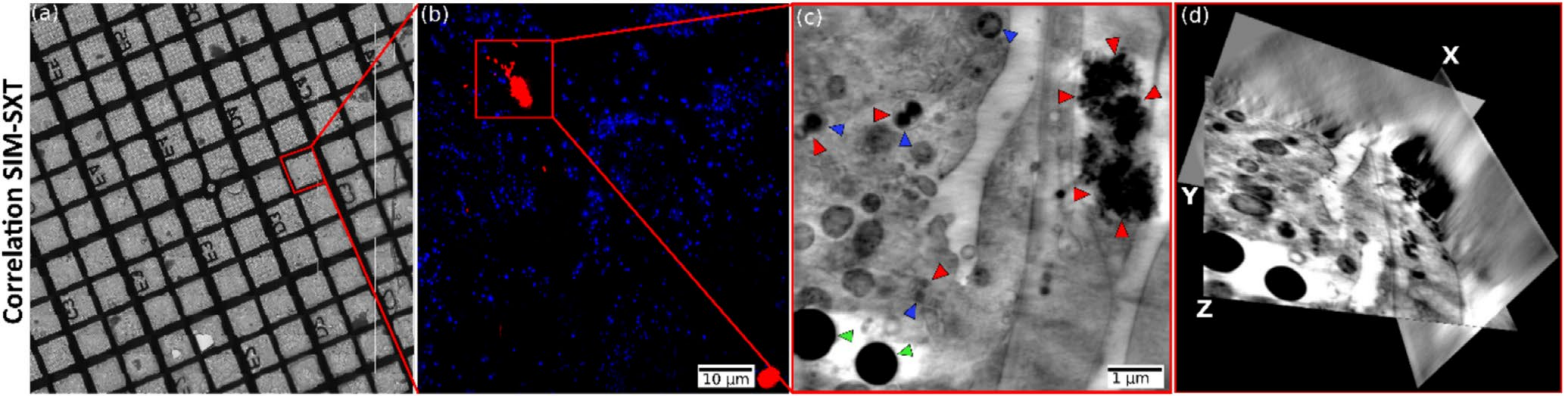
Correlative cryogenic CLXT workflow enables the unambiguous fluorescent IOH‐NP localization within its mesoscale ultrastructural context at high‐throughput. (a) Cryogenic brightfield image of the EM grid, highlighting a region of interest, corresponding to the zoomed‐in cryo‐SIM image in (b), showing IOH‐NPs (red) and lysosomes (blue, LysoTracker), with a boxed area indicating the region of interest imaged by SXT in (c) showing IOH‐NPs (red arrowheads), endolysosomal vesicles (blue arrowheads), and lipid droplets (green arrowheads). (d) Orthoslices of the volumetric data where the IOH‐NPs can be seen within the cell.

Figure 3 illustrates a correlative cryogenic light and X‐ray tomography (CLXT) workflow for unambiguous identification and localization of fluorescent IOH‐NPs within their mesoscale ultrastructural context. Panel (a) shows a cryogenic brightfield overview image EM grid, capturing a large field of view to facilitate sample navigation and selection of ROI. The red box highlights a specific cell that was targeted for higher‐resolution imaging. (b) The magnified region from panel (a) is shown as a cryo‐SIM image. IOH‐NPs are visualized in red, while lysosomes are labeled in blue using LysoTracker dye. This fluorescence data provides molecular specificity and guides targeted SXT imaging. The red box indicates the area that is subsequently imaged using SXT in panel (c). Panel (c) Soft X‐ray tomogram of the selected region reveals cellular ultrastructure at mesometric resolution. IOH‐NPs appear as dense dark aggregates (marked with red arrowheads), clearly localized within vesicular compartments. Endolysosomal vesicles (blue arrowheads) and lipid droplets (green arrowheads) are also distinguishable, enabling correlation between molecular identity and ultrastructural morphology. (d) An orthogonal slice view (orthoslice) of the three‐dimensional SXT volume is presented, showing the spatial distribution of IOH‐NPs within the cellular context. The X, Y, and Z axes denote the orientation of the 3D dataset, facilitating comprehensive analysis of particle localization in three dimensions.

**FIGURE 4:**
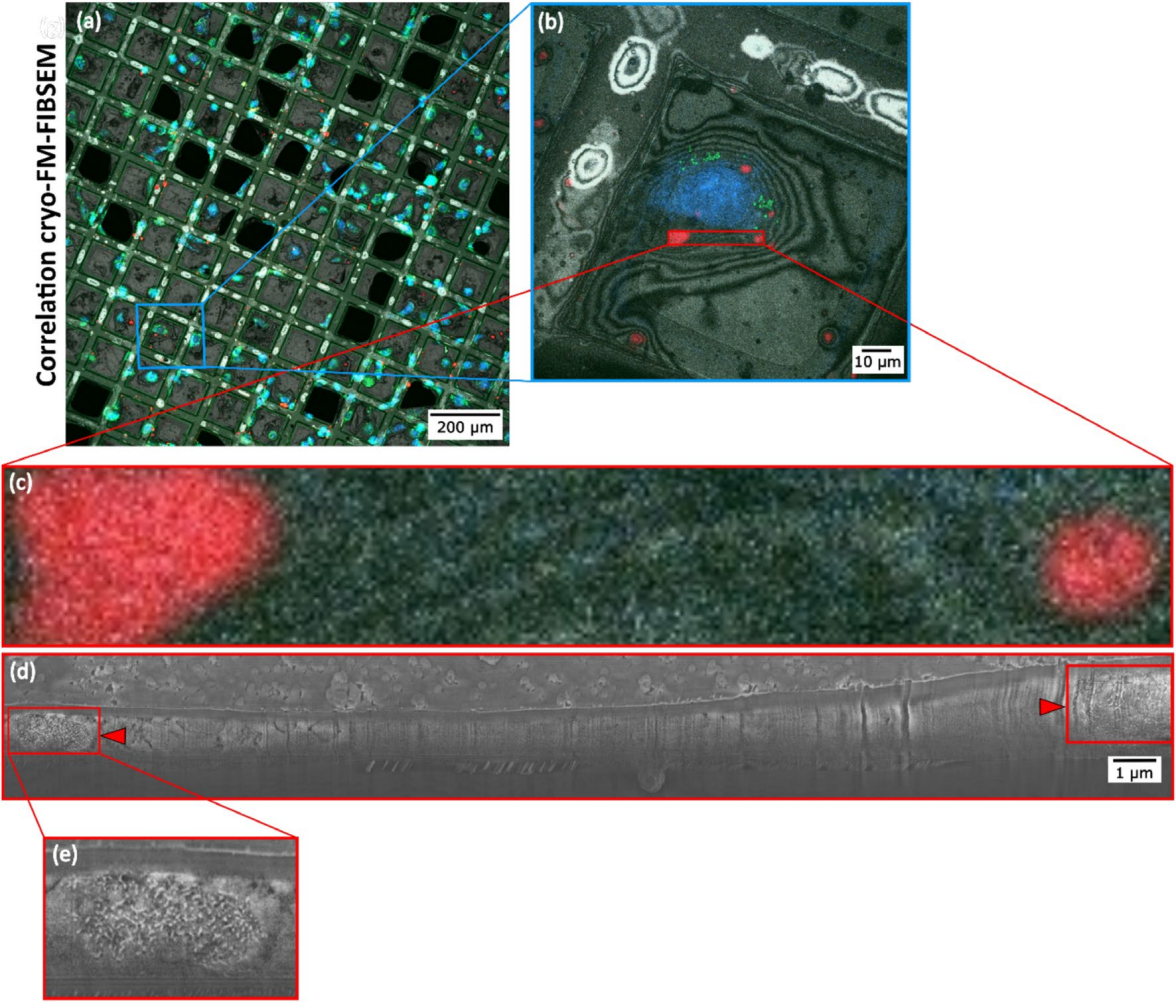
Correlative cryogenic CLEM workflow enables high‐resolution mapping of IOH‐NP localization and subcellular ultrastructure in murine breast cancer cells. (a) FM tile scan overview of an EM‐grid region showing multiple cells with IOH‐NP accumulation (red), nuclear stain (blue, Hoechst), and lipid droplets (BODIPY, green). (b) Higher magnification Airyscan FM image of a single cell with IOH‐NPs in red, lipid droplets in green, and nuclei in blue. (c) Enlarged FM section of IOH‐NPs inside the cell. (d) FIBSEM cryo‐section, showing the cell morphology after site‐specific milling near an IOH‐NP‐containing region (red arrowheads). In cryogenic contrast, based on the correlation with the Airyscan FM image in (c), IOH‐NPs can be unambiguously identified as white dots. (e) Zoomed‐in view of the NP cluster.

Figure 4 illustrates a correlative cryo‐CLEM workflow designed to precisely map the subcellular localization of IOH‐NPs within murine breast cancer cells. Panel (a) A Widefield FM tile scan of an EM grid is shown, highlighting a large number of cells. IOH‐NPs appear in red, cell nuclei are stained blue with Hoechst dye, and lipid droplets are labeled in green using BODIPY. This overview facilitates the identification and targeting of ROIs for subsequent high‐resolution imaging. (b) A magnified Airyscan FM image of a selected cell from panel (a) provides enhanced resolution. Panel (c) A further zoom‐in of the fluorescence signal confirms the intracellular localization of IOH‐NPs. The high‐intensity red signal indicates NP clusters within the cellular context, supporting precise targeting for correlative cryo‐FIBSEM imaging. (d) A cryogenic FIBSEM lamella image shows the ultrastructure of the cell after site‐specific milling through the region containing IOH‐NPs (as determined from panel c). The red arrowheads mark the NP‐containing area. In the cryogenic contrast conditions used here, IOH‐NPs appear as bright (white) dense particles, distinguishable from the surrounding cytoplasmic matrix. (e) A magnified view of the IOH‐NP cluster from panel (d) reveals its fine ultrastructural features. This zoomed‐in image confirms the localization and morphology of the NP aggregate at nanometric resolution.

Figures 3 and 4 highlight the correlation strategy that allowed for the visualization of engulfed IOH‐NP in each imaging modality, and the correlative workflow shows that cryogenic microscopy is a powerful tool for studying NP uptake. Cryogenic SXT and FIBSEM also revealed the ultrastructure of the cancer cells at a high enough resolution and contrast to be quantified. Importantly, IOH‐NP and cell organelles were unambiguously identified by correlating the SXT and FIBSEM with the FM datasets. Based on this correlation with FM, all cryogenic imaging modalities proved valuable microscopy techniques to localize IOH‐NPs recognizably and study their uptake in vitro quantitatively.

### 3.2 NPs Enter Cells Within 2 h

**FIGURE 5:**
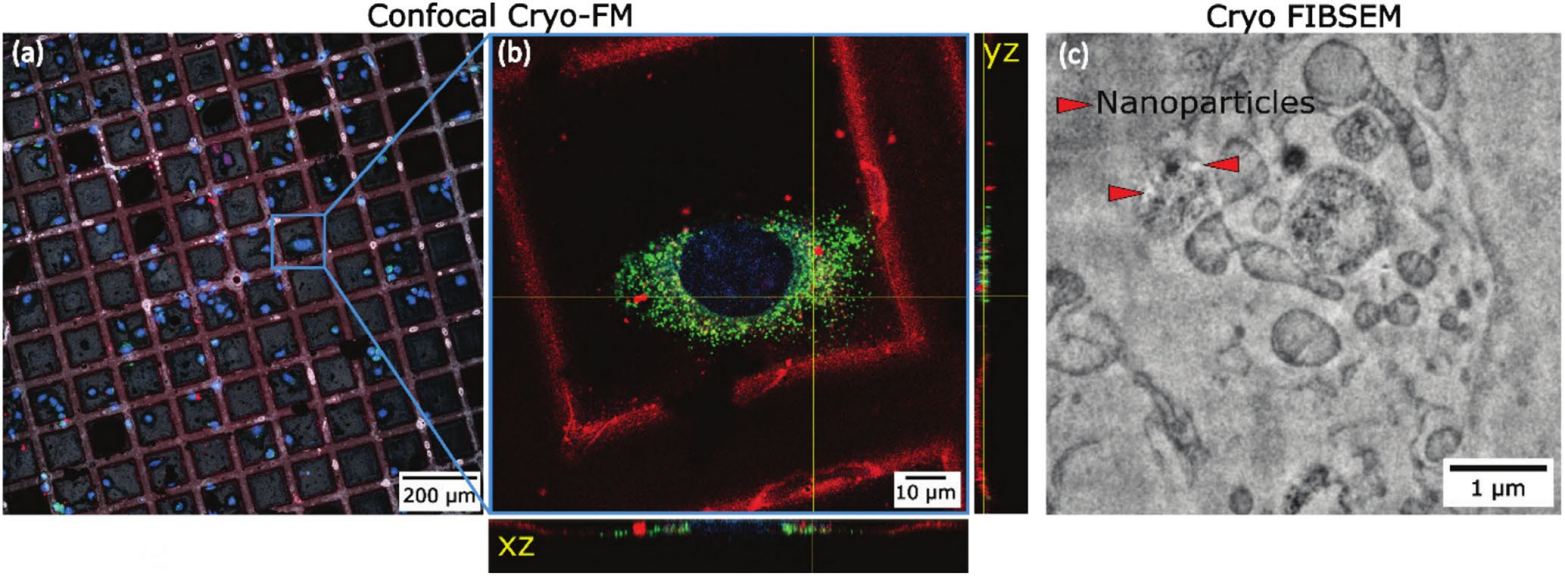
Cryogenic FM maximum intensity projection (MIP), orthoslice, and FIBSEM cross section of cells 2 h after incubation with IOH‐NPs. (a) Low‐magnification FM overview image showing a cryo‐preserved grid with Hoechst‐stained nuclei (blue). (b) High‐resolution Airyscan image highlighting a single cell with engulfed IOH‐NP clusters (red), lipid droplets (green BODIPY), and nucleus (blue, Hoechst). Engulfment is confirmed based on the confocal orthoslices of the cryo‐FM dataset in X‐Z (c). (d) FIBSEM images of H8N8 breast cancer cells show IOH‐NP uptake into vesicular organelles (probably endolysosomes) after 2 h (red arrowheads). As established through correlation (see Figure 4), in images acquired using cryogenic FIBSEM, IOH‐NPs appear white.

Figure 5 illustrates the intracellular fate of IOH‐NPs at early time points following uptake by cancer cells. We show that, after 2 h of IOH‐NP incubation, our cryogenic microscopy approach reveals IOH‐NP uptake into the breast cancer cells across scales. The cryo‐confocal FM image shows engulfed clusters of fluorescently labeled IOH‐NPs (red), BODIPY‐stained lipid‐rich organelles (green), and Hoechst‐labeled nuclei (blue) in (b). The uptake and engulfment of the IOH‐NP after 2 h are illustrated by the corresponding orthoslices of the cryo‐FM dataset. (c) The image acquired by cryo‐FIBSEM at higher magnification confirms that IOH‐NPs are found within membrane‐bound vesicles, likely endolysosomal compartments (cp. Figure 4), specifically the zoomed‐in view of NP clusters in cryo‐FIBSEM. The consistent vesicular encapsulation supports the interpretation that internalized IOH‐NPs are initially trafficked via the endocytic pathway. The SXT orthoslices in Figure 3d confirm IOH‐NP clusters inside the cancer cell (dark electron‐dense features marked by red arrowheads) enclosed within vesicular structures, which are morphologically consistent with endolysosomal vesicles.

Together, these multimodal cryo‐CLXT and cryo‐CLEM datasets confirm the rapid internalization of IOH‐NPs within 2 h of exposure and illustrate their intracellular fate by canonical endo‐lysosomal trafficking. The integration of cryo‐FM with cryo‐SXT or cryo‐FIBSEM offers a robust platform for tracking nanomaterials in situ and assessing their intracellular dynamics with high spatial and contextual resolution.

### 3.3 NPs Are Engulfed by Endolysosomes With Increasing Endolysosomal Sizes Over Time

**FIGURE 6:**
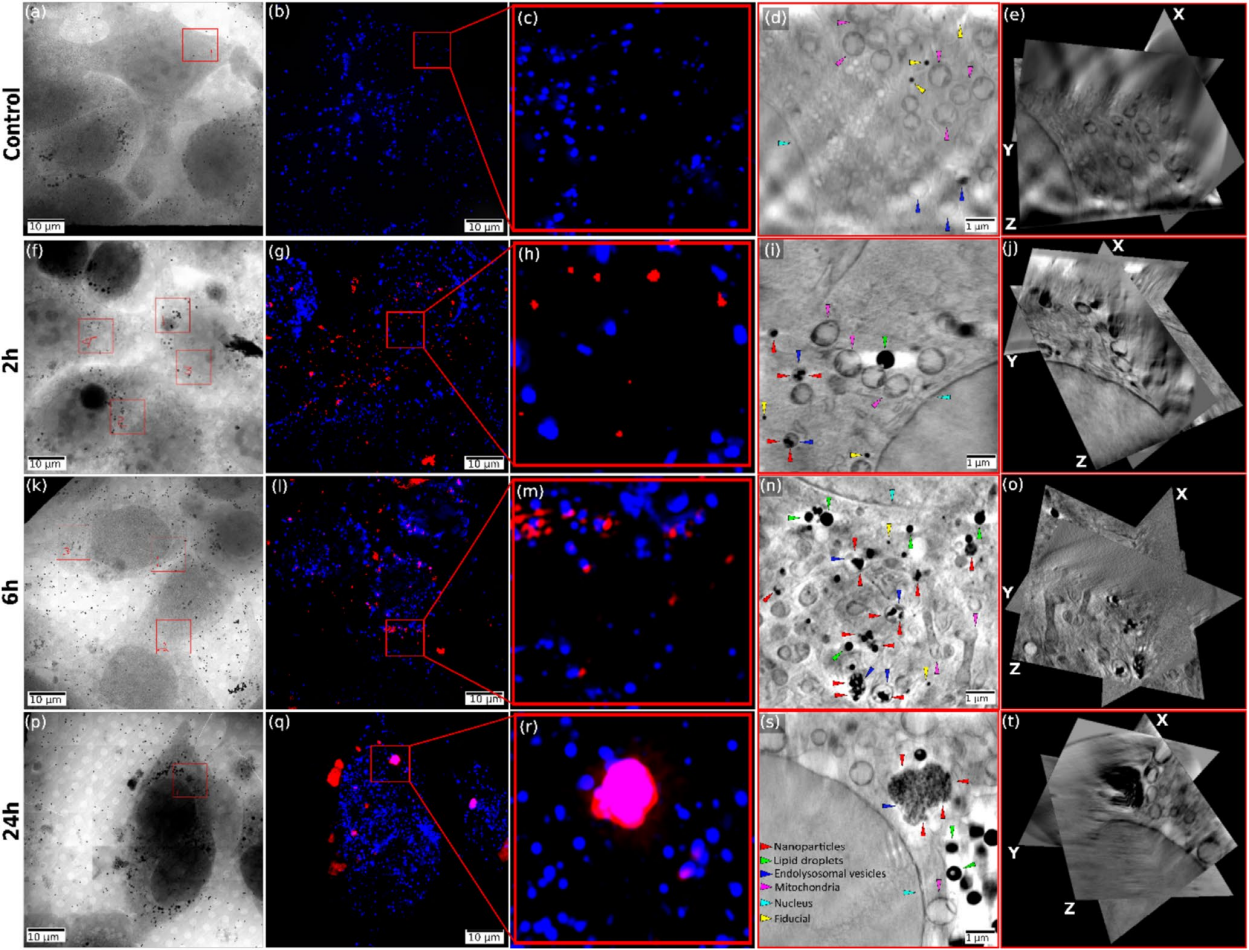
The time‐dependent accumulation of IOH‐NPs in endolysosomes is associated with increased endolysosomal size. Panels (a)–(e): Control (without IOH‐NPs). Yellow arrowheads show fiducials, gold markers which are used for high‐accuracy correlation across modalities (cp. Methods section). (f) and (j): After 2 h of IOH‐NP incubation. (f) X‐ray mosaic image showing the intracellular structures and the region of interest. (g) Cryo‐SIM image showing endolysosomes (blue) stained with LysoTracker and fluorescently labeled IOH‐NPs (red) with little overlap (pink). Region of interest marked for high‐resolution analysis (red box). (h) Zoomed in view on the SIM region of interest for better NPs visualization. (i) SXT image revealing intracellular vesicles and IOH‐NP localization (red arrowheads) based on the correlation with the cryo‐SIM image with small endolysosomal sizes. (j) Orthoslices of the soft X‐ray tomogram. Panels (k)–(o): 6 h of NP incubation. (k) Projection image with region of interest indicating regions of dense IOH‐NP accumulation. (l) Cryo‐SIM shows an increased clustering of IOH‐NP around the nucleus and within lysosomes (pink indicating colocalization of endolysosomal vesicles and NPs). (m) Zoomed in view on the SIM region of interest. (n) SXT image showing more pronounced endolysosomal structures with bigger vesicle density and size. (o) Orthoslices of the soft X‐ray tomogram. Panels (p)–(t): 24 h of IOH‐NP incubation. (p) Projection image highlighting large vesicular structures. (q) Cryo‐SIM revealing massive IOH‐NP accumulation and co‐localization with endolysosomes (pink). (r) Zoomed in on the SIM region of interest. (s) SXT reveals enlarged lysosomes heavily loaded with IOH‐NPs (red arrowheads) and surrounding organelles, suggesting advanced maturation of endo‐lysosomal compartments. (t) Orthoslices of the soft X‐ray tomogram.

Figure 6 presents a time‐course analysis of IOH‐NP uptake and intracellular trafficking in breast cancer cells using the cryo‐CLXT workflow. The panels illustrate the progressive intracellular accumulation of IOH‐NPs over time and their increasing colocalization with lysosomes, suggesting a time‐dependent enhancement in endolysosomal engagement and potential lysosomal activation. For comparison, a control sample is shown, panels (a)–(e), where no IOH‐NPs were added to the cells. (a) shows an SXT mosaic overview and (b) the corresponding cryo‐SIM image, where lysosomes (blue) are visualized. (c) provides a zoomed‐in view of the SIM region of interest for visualization. The magnified SXT image in (d) shows various organelles like mitochondria (magenta arrowheads), endolysosomal vesicles (blue arrowheads), as well as the nucleus with its nuclear membrane (cyan arrowhead).

Panels (f)–(j) represent the earliest time point (2 h) postincubation. In the SXT overview image (f), numerous vesicles containing electron‐dense IOH‐NPs can be identified, and their spatial distribution is mapped to the corresponding cryo‐SIM image (g), where IOH‐NPs (red) and lysosomes (blue) are visualized. A magnified analysis of the boxed regions reveals that the IOH‐NPs are localized within small, membrane‐bound vesicles (h), which are morphologically consistent and colocalize with the stained lysosomes. Importantly, the vesicles containing IOH‐NPs appear relatively small and are dispersed throughout the cytoplasm. Various organelles—including endolysosomes and lipid droplets (green arrowheads) can be clearly resolved and annotated based on their morphology and contrast in SXT (i) and based on the correlation with the SIM images. (j) An orthogonal slice view (orthoslice) of the three‐dimensional SXT volume is presented, showing the spatial distribution of IOH‐NPs within the cellular context. The X, Y, and Z axes denote the orientation of the 3D dataset, facilitating comprehensive analysis of particle localization in three dimensions.

At the intermediate time point of 6 h (panels (k)–(o)), a notable increase in the number and size of vesicles harboring IOH‐NPs is observed. The corresponding cryo‐SIM image (l) and zoomed‐in view of NPs (m) show more extensive colocalization of the IOH‐NP signal with endolysosomal vesicles. SXT (n) and orthoslices (o) confirm that these vesicles have increased in size and appear more densely packed, indicative of dynamic endosomal processing and lysosomal engagement. By the latest time point of 24 h (panels (p)–(t)), there is a pronounced accumulation of IOH‐NPs in large lysosomal compartments. The cryo‐SIM image (q) and zoomed‐in view of NPs (r) reveal a strong and condensed NP signal in distinct endolysosomal regions, and the high‐resolution SXT image (s) and orthoslices (t) show the corresponding densely packed vesicles with heterogeneous internal content within the endolysosomes. Many of the vesicles are considerably enlarged and contain aggregates of IOH‐NPs, consistent with active lysosomal sequestration. These observations indicate a time‐dependent shift in IOH‐NP localization from dispersed endocytic compartments to mature lysosomes that increase in size over time.

Collectively, this figure demonstrates the temporal evolution of IOH‐NP trafficking within cancer cells, characterized by increased lysosomal involvement, vesicular enlargement, and intracellular redistribution. The progressive association of IOH‐NPs with lysosomes over time likely reflects both enhanced uptake and intracellular sorting mechanisms. These findings underscore the dynamic nature of IOH–NP–lysosome interactions and reinforce the value of correlative cryogenic imaging approaches, such as cryo‐SIM combined with SXT, for high‐resolution, time‐resolved mapping of nanomaterial behavior in intact cells.

**FIGURE 7:**
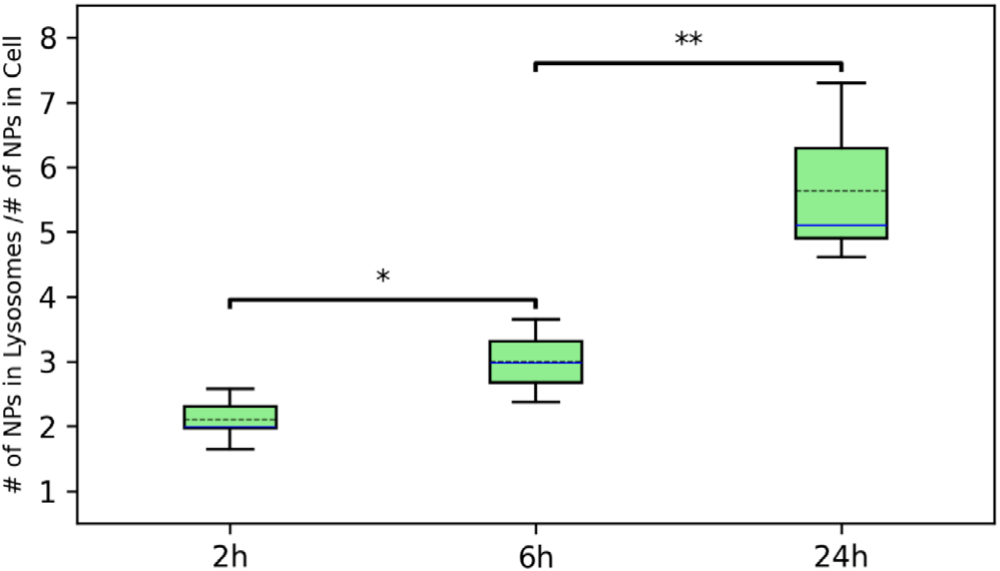
Quantification of intracellular IOH‐NPs and lysosomal co‐localization over time based on cryogenic CLXT. Box plots show the percentage of IOH‐NPs localized within lysosomes (green) at 2, 6, and 24 h postincubation as compared to the cell volume. Quantification was performed based on cryo‐SXT datasets. The solid blue lines represent the median, and the dashed black lines indicate the mean values. The *p* value for 2 and 6 h for the percentage of IOH‐NP in lysosome is 0.010 (indicated by *), and the *p* value for 6 and 24 h is 0.008 (indicated by **). *n* = 5 for 2 h, *n* = 5 for 6 h, and *n* = 7 for 24 h. The IOH‐NP size is (60 × 60 nm).

To evaluate the temporal dynamics of IOH‐NP trafficking within cancer cells, we quantified the IOH‐NP distribution based on the CLXT datasets at 2, 6, and 24 h postincubation, as described in the methods section. As shown in Figure 7, the proportion of IOH‐NPs localized within endolysosomes, highlighted in the box plots, increased significantly with time. This trend is consistent with our visual inspections, indicating progressive intracellular trafficking of IOH‐NPs into the endolysosomal pathway. Consistent with this observation, cryo‐SXT imaging revealed a time‐dependent increase in both the number and size of lysosomal compartments.

## 4 Discussion

### 4.1 Cryogenic Microscopy Across Modalities

We demonstrate that combining cryogenic correlative SIM‐SXT and cryogenic correlative FM‐FIBSEM workflows enables the quantitative study of IOH‐NP uptake into murine breast cancer cells in 3D, making it a powerful tool for future studies aiming to identify structures in close‐to‐native cells unambiguously. While the cryo‐CLXT workflow allows for high throughput with mesoscale resolution, the cryo‐CLEM workflow allows for the visualization of single IOH‐NP at nanometer resolution at the expense of long data acquisitions of more than a day per cell. The soft X‐ray “water window” energy range between the carbon *K* edge at 0.28 keV and the oxygen *K* edge at 0.54 keV provides minimum dose imaging for specimens in ice layers up to about10–20 μm thick, with doses reaching about 10^6^ Gray (Le Gros et al. 2005). This dose does not cause appreciable structural changes to the sample at the resolution scale of the SXT but might lead to considerable damage at the nanometer scale. By applying a bi‐headed workflow and separating it into a cryo‐CLEM and a cryo‐CLXT branch, we were able to use SXT as an end imaging technique without considering induced radiation damage to high‐resolution cellular structures, as would be the case for a correlative light, electron, and X‐ray microscopy (CLEXM) workflow on the same cell.

While the cryogenic contrast is enough in SXT to identify both IOH‐NPs and cellular compartments, the unambiguous localization of IOH‐NPs in cryogenic FIBSEM datasets is only possible based on the registration with cryo‐FM datasets that identified NPs in corresponding ROI. For this, ideally, a voxel‐to‐voxel correspondence between FM and SXT should be achieved. Due to a more than 5‐fold difference in pixel sizes (SXT about 30 nm, cryo‐FM > 250 nm), the unambiguous identification of these single particle sightings is hampered, and we cannot exclude that artifacts in the contrast or sample preparation cause them. This makes the necessity of cryogenic super‐resolution FM approaches evident, which achieve single NP resolutions below 50 nm, such as cryo‐SOFI (Moser et al. 2019).

In addition, automation of the process under cryogenic conditions, including automated segmentation and quantification of cell organelles, is challenging due to the much‐reduced SNR ratio as compared to the already low SNR at room temperature. While several solutions have been suggested for automatically quantifying FIBSEM datasets at room temperature based on machine learning (Nešić et al. 2024), these approaches have not yet been tested for cryogenic FIBSEM and SXT datasets. The automation of the quantification process is additionally hampered since EM visualizes the ultrastructural details and rich contextual information based on protein/lipid or stain‐density gradients. This includes the target organelles and the broader membrane‐delineated context of the intracellular environment, increasing the difficulty of specific feature extraction. Besides, the interpretation of cryogenic FIBSEM datasets is hampered due to the nonisotropic data acquisition.

Regarding cryogenic sample preparation, it is essential to achieve optimal cryogenic fixation without ice crystal formation to preserve the near‐native state of single cells. Preparing samples for cryogenic microscopy is extremely challenging and should ideally be conducted by experienced scientists. Key parameters to optimize include the cooling rate, blotting time (for plunge freezing), and sample thickness to ensure proper vitrification without ice formation. While plunge freezing is rapid and suitable for thin samples, it poses a risk of insufficient cooling in thicker specimens, which can cause artifacts from ice crystals. Conversely, high‐pressure freezing provides better vitrification for thicker samples by increasing the cooling rate under high pressure, but it requires specialized equipment and careful handling to prevent sample damage. We did not observe ice crystal formation and believe that we successfully vitrified murine breast cancer cells at scales accessible with the imaging techniques presented, using plunge freezing.

In terms of correlative microscopy, we performed cryo‐SXT and cryo‐FIBSEM on different cells, which allowed us (based on the correlation with cryo‐FM) to gather complementary information. SXT and FIBSEM could also be combined in a correlative manner using CLEXM, with FIBSEM at the end of the workflow due to its destructive nature. Performing cryo‐FIBSEM after cryo‐SXT within a CLEXM workflow would enable high‐resolution zoom‐in on specific cellular regions identified in the lower‐resolution tomographic data. However, a CLEXM workflow can present several challenges: accurately correlating SXT and FIBSEM datasets is technically demanding because of differences in resolution, contrast mechanisms, and field of view. Current software tools for multimodal dataset alignment are limited, and manual correlation is often necessary, which can be time‐consuming and prone to errors. The physical transfer of cryogenic samples between instruments also requires careful handling to prevent devitrification or ice contamination. We show here that combining cryogenic CLEXM and CLEM workflows enables complementary visualization of NP uptake at both mesoscale and nanometer resolutions, offering a versatile strategy for quantitative, structure–function analysis in near‐native cellular environments.

### 4.2 NP Uptake

In line with others (Akhtar and Zuhair 2025; Ribeiro et al. 2025) and own observations (Ischyropoulou et al. 2023; Notter et al. 2024) using different types of IOH‐NPs, we observed IOH‐NP uptake into cancer cells and transport into endolysosomes within 2 h. While FM provided the necessary data to find ROIs and unambiguously identify IOH‐NPs, the FIBSEM provided a zoomed‐in, detailed view of these regions. SXT enabled high workflow throughput to assess NP distributions with significant quantitative statistics. Despite the imaging advantages, the current quantification strategy relies on blob detection using the Difference of Gaussian (Blob‐DoG) function in the scikit‐image Python package. To fully leverage the high‐throughput potential of cryo‐SXT, more advanced and automated image analysis workflows—potentially incorporating machine learning—are urgently needed to handle and interpret data from large cell populations efficiently and reproducibly.

Besides, further studies need to be conducted at different time points to illustrate the pathway of these IOH‐NPs from cell entrance to cell clearance or dissolution within the murine cancer cells. Interesting time points include 30 min, 1, 5, 12, 18, and 48 h, and would provide a more complete temporal profile of IOH‐NP dynamics from initial cellular uptake to endosomal trafficking, lysosomal accumulation, and potential exocytosis or final dissolution for drug release. These studies could reveal insights into the mechanisms of cellular processing, clearance, or retention of IOH‐NPs, which are critical for both safety assessment and therapeutic efficacy.

The next steps involve loading the IOH‐NPs with chemotherapeutics to examine how the drug is released and combining cryo‐microscopy with molecule‐specific imaging techniques, such as mass spectrometry imaging (MSI). This can help determine the spatial location of both the NP as a drug carrier and the released drug within different organelles, including endosomal escape and the drug entering the nucleus, offering a detailed view of drug delivery, release, and activity at the subcellular level.

## 5 Conclusion

This paper highlights the combination of cryogenic FM‐FIBSEM (cryo‐CLEM) and cryogenic SIM‐SXT (cryo‐CLXT) workflows as powerful tools to quantitatively assess and study NP uptake into murine breast cancer cells across different scales. While CLXT provided high‐throughput mesoscale analysis of NP uptake, CLEM enabled high‐resolution visualization of single IOH‐NPs within cell organelles. For both CLEM and CLXT, cryogenic FM was used to identify ROI and clearly distinguish IOH‐NPs, the nucleus, and lipid droplets in CLEM, and IOH‐NPs and lysosomes in CLXT. The high contrast in cryogenic imaging allows for easy identification of cellular compartments by visual inspection; certainty is achieved by correlating cryogenic FM with corresponding staining. Based on CLXT datasets, the density of IOH‐NPs was measured within cells at different (cryo) fixation time points, especially within specific organelles. Additional labeling of lipid droplets with BODIPY enabled rough alignment of cryogenic FM data with FIBSEM datasets, as they were easily detectable across all modalities without extra staining. Lipid droplets are electron dense under cryogenic conditions in FIBSEM and exhibit high absorption in SXT due to functioning within the water window. Moreover, cryogenic microscopy greatly reduced sample preparation time from about a week to a few hours. Throughput was further increased by incorporating cryo‐SXT into the workflow, efficiently handling the large number of samples necessary for biomedical research on inherently diverse cancer cells.

## Author Contributions

**Pavitra Sokke Rudraiah:** conceptualization, investigation, writing – original draft, writing – review and editing, visualization, funding acquisition, formal analysis, methodology, data curation. **Louisa Herbsleb:** investigation, writing – original draft, writing – review and editing, formal analysis, data curation, methodology. **Michaela Salakova:** investigation, data curation, methodology. **Henriette Gröger:** investigation, resources. **Anna Maria Steyer:** investigation, methodology, visualization, formal analysis, resources. **Frauke Alves:** conceptualization, investigation, writing – review and editing, resources, validation. **Claus Feldmann:** conceptualization, investigation, resources, writing – review and editing. **Andreas Walter:** conceptualization, funding acquisition, writing – review and editing, writing – original draft, supervision, project administration, validation, investigation.

## Conflicts of Interest

The authors declare no conflicts of interest.

## Data Availability

The data that support the findings of this study are available from the corresponding author upon reasonable request.
